# ERK Signaling in Thyroid Cancer: Lineage Suppression, Epigenetic Reprogramming, and Theranostic Reversal

**DOI:** 10.3390/cancers18162697

**Published:** 2026-08-20

**Authors:** Sara Ashtari, Mohammad M. Mehrabi, Seza A. Gulec

**Affiliations:** 1 Faculty of Medicine, Tehran University of Medical Sciences, Tehran 1416753955, Iran; sara.ashtari2@gmail.com (S.A.); or mehrabi.mm.md@gmail.com (M.M.M.); 2 Miami Cancer Research Center, Miami, FL 33180, USA

**Keywords:** MAPK, ERK signaling, thyroid cancer, functional differentiation, radioiodine-refractory differentiated thyroid cancer, sodium/iodide symporter, redifferentiation therapy, theranostics, differentiated thyroid carcinoma

## Abstract

Differentiated thyroid carcinoma is a morphologic diagnosis, not a direct measure of preserved thyroid function. Although most papillary, follicular, and oncocytic thyroid carcinomas retain architectural or cytologic features of follicular-cell derivation, a clinically important subset loses the molecular machinery required for effective iodine transport, organification, and radioiodine therapy. This functional divergence is central to the biology of radioiodine-indifferent and radioiodine-refractory thyroid cancer. This review examines the role of oncogenic *MAPK/ERK* signaling as a principal regulator of thyroid lineage suppression and therapeutic failure. Aberrant activation of the *RAF–MEK–ERK* axis, most prominently through *BRAF^V600E, RAS* alterations, and receptor tyrosine kinase fusions, does more than promote proliferation. It reshapes follicular-cell identity by repressing thyroid-lineage transcriptional programs and silencing iodine-handling genes, including *SLC5A5/NIS, TPO, TG, TSHR, SLC26A4*, and related components of the iodine metabolic transcriptome. This repression is mediated through coordinated transcriptional, epigenetic, and post-transcriptional mechanisms involving lineage transcription factors such as *PAX8, NKX2-1*, and *FOXE1*; chromatin-modifying programs including histone deacetylation and *PRC2/EZH2*-associated repression; DNA methylation; and non-coding RNA networks. Importantly, *ERK*-driven functional dedifferentiation is not always a fixed terminal state. Preclinical models and early clinical trials demonstrate that pharmacologic inhibition of the *MAPK* pathway can restore iodine avidity in selected radioiodine-refractory tumors, creating the theranostic basis for redifferentiation therapy. Iodine-124 PET/CT and lesion-level dosimetry have shown that restored iodine uptake can be measured diagnostically and then exploited therapeutically with iodine-131. However, clinical translation remains limited by inter- and intratumoral heterogeneity, incomplete durability of response, adaptive pathway reactivation, persistent epigenetic repression, tumor microenvironmental influences, and the absence of standardized dosimetric thresholds. We propose that advanced follicular-cell-derived thyroid cancers should be understood along a functional differentiation continuum rather than through morphology alone. Within this framework, radioiodine refractoriness reflects not merely treatment failure, but a therapeutically interrogable state of lineage suppression. Refinement of redifferentiation therapy will require genotype-informed patient selection, functional imaging, standardized dosimetry, rational combination strategies, and prospective trials capable of distinguishing true restoration of radioiodine therapeutic efficacy from the antiproliferative effects of kinase inhibition alone.

## Simple Summary

Thyroid cancers can appear well differentiated under the microscope yet lose the cellular machinery needed to absorb radioactive iodine, making this treatment less effective. This review examines how persistent activation of a cell-signaling pathway called ERK can suppress the normal thyroid-cell program, alter gene regulation, and reduce the ability of cancer cells to handle iodine. It also evaluates experimental and clinical attempts to reverse this process with targeted drugs so that tumors may regain sensitivity to radioactive iodine. Current evidence shows that such “redifferentiation” is biologically possible in selected tumors, but responses vary and restored iodine uptake does not always result in meaningful tumor control. Future progress will depend on better biomarkers, improved selection of patients and individual tumor lesions, standardized imaging and radiation-dose assessment, and clinical trials that distinguish restored radioactive iodine sensitivity from the direct anticancer effects of targeted therapies.

## 1. Introduction

Thyroid cancer (TC) is the most common endocrine malignancy, with increasing global incidence and persistently low disease-specific mortality in most patient populations [1,2,3]. This apparent paradox reflects the broad biological spectrum of follicular cell-derived TCs. Many tumors are indolent and highly curable, whereas a smaller but clinically decisive subset progresses to recurrent, metastatic, and radioiodine-refractory (RAIR) disease [4].

Differentiated thyroid carcinoma (DTC) refers to papillary, follicular, and oncocytic (Hürthle-cell) carcinomas, characterized by morphological features typical of follicular epithelial origin [5]. However, in a theranostic context, this term may be misleading, as a tumor can appear differentiated microscopically yet lack the necessary mechanisms for effective iodine uptake. This distinction is crucial in thyroid oncology, as radioiodine (RAI) therapy relies on an intact iodine-handling program involving several key components [6]. When this program is impaired, tumors may remain morphologically differentiated but not respond to RAI treatment, leading to a state known as RAIR-TC [7,8]. Current strategies for advanced TC include risk-adapted surgery, selective RAI use, surveillance, local therapies, and systemic kinase inhibition [9,10]. While multikinase inhibitors and targeted therapies can slow disease progression, they often do not restore full thyroid function [11]. Redifferentiation therapy seeks to reactivate the suppressed iodine-handling mechanisms, enabling RAI to be used effectively as a targeted therapy [12,13].

The mitogen-activated protein kinase (*MAPK*) pathway, especially extracellular signal-regulated kinase 1/2 **(***ERK1/2*) signaling, links oncogenic transformation to lineage suppression and therapeutic reversal [10,14]. Alterations in *BRAF, RAS, RET*, and *NTRK* converge on *RAF–MEK–ERK* signaling, but impact thyroid differentiation differently [15]. The Cancer Genome Atlas (TCGA) framework highlighted that driver genotypes correlate with histology and transcriptional states [16]. Within *BRAF*-mutant cases, varying thyroid differentiation levels have clinical significance [17,18]. This review treats *ERK* signaling as a lineage-regulating system rather than just a mitogenic cascade. In TC, sustained *ERK* activation disrupts normal follicular-cell functions and contributes to RAI resistance, yet selected models show potential for reversibility, supporting theranostic redifferentiation.

The concept of functional misdifferentiation may help resolve the ambiguity embedded in the conventional term DTC. These tumors are not necessarily undifferentiated in the morphologic sense; rather, they may be misaligned from the normal functional program of the thyroid follicular cell. This distinction is more than semantic. It determines whether RAI therapy is biologically plausible, whether redifferentiation therapy is rational, and whether advanced TC should be treated according to morphology alone or according to measurable preservation, loss, and potential restoration of thyroid lineage function.

## 2. Thyroid Functional Differentiation as a Distinct Biological Axis

The term DTC is traditionally anchored in histopathology, denoting tumors that retain architectural or cytologic evidence of follicular-cell derivation [19]. However, in advanced thyroid oncology, morphologic differentiation and functional differentiation are not interchangeable concepts. A papillary, follicular, or oncocytic TC may remain morphologically recognizable as follicular-cell-derived while losing the molecular program required for iodide uptake, organification, hormone biosynthesis, and RAI responsiveness. This distinction is clinically consequential because RAI efficacy depends less on microscopic appearance than on the preservation, suppression, or recoverability of thyroid-specific function. Accordingly, thyroid functional differentiation should be conceptualized as a distinct biological axis that intersects with, but is not reducible to, histologic classification [16,20].

The foundation of this axis lies in thyroid organogenesis and follicular-cell lineage commitment. Thyroid follicular identity is established through a coordinated transcriptional network centered on *NKX2-1, PAX8, FOXE1*, and *HHEX*, whose combined activity specifies thyroid progenitor survival, migration, follicular organization, and terminal differentiation [21,22,23]. These transcription factors do not function as isolated lineage markers; rather, they operate as an interdependent regulatory system that sustains the differentiated thyrocyte phenotype throughout adult life. *PAX8* and *NKX2-1* are particularly important for maintaining the expression of thyroid-specific genes, while *FOXE1* and *HHEX* contribute to developmental positioning, structural organization, and continued lineage fidelity [22,23].

At the functional level, this transcriptional network converges on the machinery of iodide metabolism and thyroid hormone biosynthesis. The sodium/iodide symporter (*NIS*), encoded by *SLC5A5*, mediates basolateral iodide uptake and represents the first indispensable step in thyroid hormone synthesis and RAI accumulation [24]. *PAX8* directly participates in *SLC5A5* transcription through binding to the *NIS* upstream enhancer, providing a mechanistic bridge between lineage transcriptional identity and iodine-handling competence [25]. After iodide entry, apical transporters such as pendrin, encoded by *SLC26A4*, facilitate iodide movement toward the follicular lumen, while thyroglobulin, thyroid peroxidase, and hydrogen peroxide–generating dual oxidase systems support organification and coupling reactions required for T3 and T4 synthesis [22,24]. Thus, functional thyroid differentiation is not defined by a single gene, but by the coordinated activity of a polarized epithelial program.

This axis is actively maintained in adult thyroid epithelium rather than passively inherited from development. Thyrotropin signaling through the TSH receptor and downstream cAMP-dependent pathways sustains the expression of thyroid-lineage and iodine-metabolism genes, including *TG*, *TPO*, *TSHR*, *SLC5A5*, and related differentiation-associated transcripts [26,27]. In adult human thyrocytes, TSH regulates differentiated gene transcription through changes in transcriptional activity rather than merely altering mRNA stability, supporting the concept that endocrine signaling continuously reinforces follicular-cell identity [27]. This tonic regulatory requirement explains why disruption of TSH responsiveness, lineage transcription factors, or chromatin accessibility can produce functional dedifferentiation even when the tumor remains morphologically classified as differentiated.

The TCGA provided a molecular framework for measuring this axis in papillary thyroid carcinoma (PTC). By integrating genomic, transcriptomic, and epigenomic data, TCGA showed that PTCs segregate into BRAF-like and RAS-like molecular states with distinct signaling output and differentiation properties [16]. Importantly, the thyroid differentiation score was derived from the expression of a 16-gene panel related to thyroid hormone biosynthesis and iodine metabolism, including *DIO1*, *DIO2*, *DUOX1*, *DUOX2*, *FOXE1*, *GLIS3*, *NKX2-1*, *PAX8*, *SLC26A4*, *SLC5A5*, *SLC5A8*, *TG*, *THRA*, *THRB*, *TPO*, and *TSHR* [16,17]. This score formalizes the idea that thyroid differentiation is measurable as a transcriptomic continuum rather than a binary histologic category.

Within this continuum, oncogenic *MAPK/ERK* signaling emerges as a central determinant of functional suppression. *BRAF^V600E*-positive PTCs show reduced expression of thyroid differentiation genes and impaired *NIS* targeting the cell membrane, providing a mechanistic basis for the association between *MAPK* activation and reduced RAI avidity [28]. Experimental inhibition of the *BRAF/MEK/MAPK* pathway can restore expression of iodide-metabolizing genes in thyroid cells expressing mutant *BRAF*, supporting the interpretation that at least part of this functional loss is pathway-dependent rather than purely irreversible [29]. Additional mechanistic work indicates that *BRAF^V600E* can induce *TGF-β* signaling, which represses *NIS* expression and promotes a more invasive phenotype, further linking oncogenic *ERK* output to lineage suppression and RAI failure [30,31].

Importantly, loss of the thyroid-lineage program is neither uniform nor necessarily synchronous across individual differentiation markers. In human DTC, *NIS, TPO, TG, TSHR*, and other iodine-handling genes can be suppressed to different degrees, reflecting heterogeneous rather than all-or-none functional dedifferentiation [32]. In particular, *Tg* expression may remain detectable despite marked impairment of *NIS* expression or membrane localization and loss of radioiodine avidity. Thus, preservation of selected follicular-lineage markers, including *Tg*, should not be interpreted as evidence that the complete iodine-handling program remains functionally intact [32,33]. This dissociation further supports viewing thyroid functional differentiation as a multidimensional continuum rather than a binary state.

A clinically useful consequence of this model is that RAI refractoriness can be interpreted as a state of functional thyroid-lineage insufficiency rather than as a mere treatment-history label. The American Thyroid Association (ATA) defines RAIR disease through patterns such as absent uptake, loss of previously present uptake, heterogeneous uptake among lesions, or progression despite uptake, all of which reflect inadequate therapeutic RAI competence at the disease level [20]. This definition is compatible with the biological axis model: a lesion may be follicular-cell-derived but insufficiently differentiated for RAI to be diagnostically or therapeutically meaningful. Therefore, the key clinical question is not only whether a tumor is histologically differentiated, but also whether it retains sufficient functional machinery for iodine-based theranostics.

The functional-axis concept also explains heterogeneity within the same driver genotype. Boucai et al. showed that BRAF-mutant PTCs can be subdivided into BRAF-TDS^hi and BRAF-TDS^lo groups, with the lower-differentiation group demonstrating less preserved iodine-metabolism gene expression and less favorable clinicopathologic features [17]. This finding argues against treating *BRAF* mutation status as a uniform surrogate for differentiation state. Instead, driver genotype, *ERK* signaling amplitude, transcriptional state, epigenetic repression, and iodine-handling capacity should be integrated when assessing biological behavior and therapeutic plausibility [16,17].

The high prevalence of *BRAF^V600E* in otherwise indolent PTC provides an important qualification to a mutation-centered model of functional dedifferentiation. *BRAF^V600E* should not be interpreted as a binary surrogate for maximal *ERK* output or inevitable loss of thyroid function. BRAF-mutant tumors span a broad range of differentiation states, including BRAF-TDS^hi tumors that retain substantial thyroid-lineage expression and comparatively favorable clinicopathologic characteristics [16,17]. The phenotypic consequence of BRAF therefore depends on signaling amplitude and duration, feedback regulation, cellular context, and cooperating molecular alterations. Progression-associated events involving *TERT, TP53, PI3K–AKT* signaling, and chromatin-remodeling pathways may determine whether a BRAF-initiated tumor remains functionally differentiated or progresses toward deeply dedifferentiated, RAIR disease [34,35,36]. Thus, *BRAF^V600E* confers a biological propensity toward lineage suppression but is neither necessary nor sufficient, by itself, to define functional differentiation or clinical aggressiveness.

The reversibility of this axis in selected tumors provides the rationale for redifferentiation therapy. In *BRAF*-driven mouse thyroid cancer models, *MAPK* pathway inhibition restored thyroid-specific gene expression and RAI incorporation, demonstrating that oncogenic *ERK*-driven dedifferentiation can remain therapeutically modifiable under certain biological conditions [37]. Clinically, selumetinib increased RAI uptake and retention in a subset of patients with advanced TC, enabling subsequent therapeutic RAI administration in selected cases [38]. These studies do not imply that all RAIR tumors are reversible, but they establish that functional differentiation is a dynamic therapeutic variable rather than a fixed morphologic attribute.

## 3. ERK Signaling in Normal Physiology and Oncogenic Transformation

In thyroid follicular cells, *ERK1/2* does not act as a consistently dominant mitogenic switch. Instead, it functions as a context-dependent effector that integrates signals from thyrotropin-dependent cAMP pathways and receptor tyrosine kinases [26,39]. In non-cancerous epithelial cells, this arrangement allows for cell proliferation only under specific growth conditions, while still maintaining the differentiated functions of the thyroid [40,41]. However, in cancer, this same signaling pathway becomes a chronically active program that shapes the lineage of the cells [40,41]. The intensity and duration of this activation are closely linked to the loss of cellular differentiation, impaired iodine handling, and the progression towards more clinically aggressive disease [42,43].

In normal thyroid epithelium, TSH signaling is the primary organizer of differentiated function [44]. However, *ERK1/2* acts as an important downstream component when trophic inputs are combined. A critical synthesis of thyroid culture models concluded that TSH, through cAMP, often serves as a competence or priming signal [26]. This amplifies the activation of *MAPK* and *PI3K* induced by IGF-I, rather than acting as a uniform standalone mitogen across all systems [45].

Specifically, in FRTL-5 cells, TSH/cAMP can induce rapid and transient *ERK1/2* activation through *Rap1* and *BRAF*, independent of protein kinase A [39]. This mechanism illustrates how endocrine signaling intersects with the canonical *MAPK* cascade. Recent studies in primary human thyrocytes further clarified the biological implications of this interaction. They demonstrated that epidermal growth factor (*EGF*) promotes cell proliferation and dedifferentiation, whereas TSH, in the absence of other growth factors, supports differentiation [40]. Collectively, these findings suggest that physiological *ERK* signaling in thyroid cells is typically brief, conditional, and situated within an environment conducive to differentiation [46].

Understanding the context dependency of *ERK1/2* is crucial to grasping its role in thyroid homeostasis in some situations, while causing a loss of differentiation in others. In healthy cells, the output of *ERK* is limited because TSH-driven programs and growth factor-driven programs are not equivalent. TSH promotes the maintenance of thyroid-specific functions, while unchecked *EGFR* activity tends to drive cells toward proliferation, moving them away from their differentiated state [40,47]. Therefore, *ERK1/2* in a healthy thyroid should be seen as a regulated part of a larger signaling network, rather than as an independent growth driver [48]. This perspective helps clarify why transformation involves not just the activation of the *MAPK* pathway, but also a sustained and enforced distortion of that signaling framework.

In follicular cell-derived TC, the regulated signaling structure is often disrupted by largely mutually exclusive genetic alterations that converge on *ERK1/2* [49]. The most prominent of these alterations include *BRAF^V600E*, *RAS* mutations, and *RET* rearrangements or fusions [42,49,50]. Early experimental evidence indicated that *RET/PTC*-induced dedifferentiation is mediated through the *Y1062-SHC-RAS-MAPK* signaling pathway, demonstrating that persistent upstream activation of this pathway is sufficient to undermine the differentiated state of thyroid cells [49].

Similarly, the expression of *BRAF^V600E* in transgenic mice is enough to induce PTC that undergo dedifferentiation [50]. Furthermore, *BRAF*-driven tumor initiation in mice relies on thyrotropin receptor signaling, suggesting that early oncogenic *MAPK* activation modifies, rather than completely replaces, the thyroid’s trophic environment [50,51]. These observations align with later mechanistic syntheses, suggesting that the main TC oncoproteins all activate the receptor tyrosine kinase–*RAS*–*BRAF* axis, albeit with distinct biochemical outputs that shape the tumor phenotype and differentiation state [42].

Among the *MAPK* drivers, *BRAF^V600E* provides the clearest model of lineage suppression. In PTCs with *BRAF* mutations, expression of differentiation genes such as *NIS*, TPO, Tg, and the apical iodide transporter, as well as the TSH receptor, is lower than in *BRAF*-wild-type tumors [32]. Additionally, *BRAF*-mutant tumors show abnormal impairment of Na^+/I^ targeting the cell membrane [28].

Mechanistically, *BRAF^V600E* can induce an autocrine *TGF-β* program that represses the *NIS* and contributes to a more malignant phenotype [28]. This links oncogenic *MAPK* signaling directly to the loss of responsiveness to RAI [30,38]. Importantly, this state is pathway-dependent rather than fixed: *MEK* inhibition in advanced TC can restore RAI uptake in a clinically meaningful subset of patients, and sustained *ERK* suppression can further enhance RAI responses in *BRAF*-driven models [30,52]. Collectively, these studies place *ERK1/2* not only at the forefront of thyroid tumor cell proliferation but also at the core of the dedifferentiation program that transforms a follicular epithelial malignancy into one that is refractory to RAI treatment [28,30,38,52].

The most influential framework for connecting genotype and phenotype in PTC was established through an integrated analysis by TCGA [16]. This analysis differentiated between *BRAF*-like and *RAS*-like transcriptional states, rather than treating all *MAPK*-pathway alterations as biologically interchangeable. It was found that stronger *MAPK* pathway output correlated with lower thyroid differentiation scores (TDS), indicating that the class of driver mutation influences phenotype partly through the amplitude of signaling and feedback mechanisms within the pathway, rather than through mutation status alone [16,42].

This concept has been further refined within *BRAF*-mutant disease. A subsequent TCGA-based study identified two subgroups: BRAF-TDS^hi^ and BRAF-TDS^lo^. The lower-differentiation subgroup was characterized by larger tumors, more involved lymph nodes, a greater number of distant metastases, and a poorer response to therapy [17]. These findings suggest that *ERK*-driven TC exist on a continuum of differentiation and that even within a single genotype, the degree of thyroid lineage preservation is both biologically and clinically significant.

Clinical cohort data strengthen the idea that genotype is not just descriptive, but also predictive of phenotype. In a study involving 1,510 patients, tumor genotype was linked to distinct phenotypes and disease-related outcomes, supporting the notion that the identity of the driver mutation reflects enduring differences in tumor biology [34]. For example, *RET* fusion-positive PTC exhibited higher rates of extrathyroidal extension, multifocal disease, and distant metastases compared to tumors driven by *BRAF^V600E*^ or *RAS* mutations [53]. This underscores the fact that different *MAPK*-activating alterations are not phenotypically equivalent, despite all converging on the same signaling network. In summary, the correlation between genotype and phenotype in TC is best understood not as a simple mapping from mutation to histology, but as a nuanced relationship where the specific biochemical mechanism of *MAPK* activation influences factors such as differentiation, invasion, metastatic potential, and therapeutic response [34,42,53].

**Figure 1:**
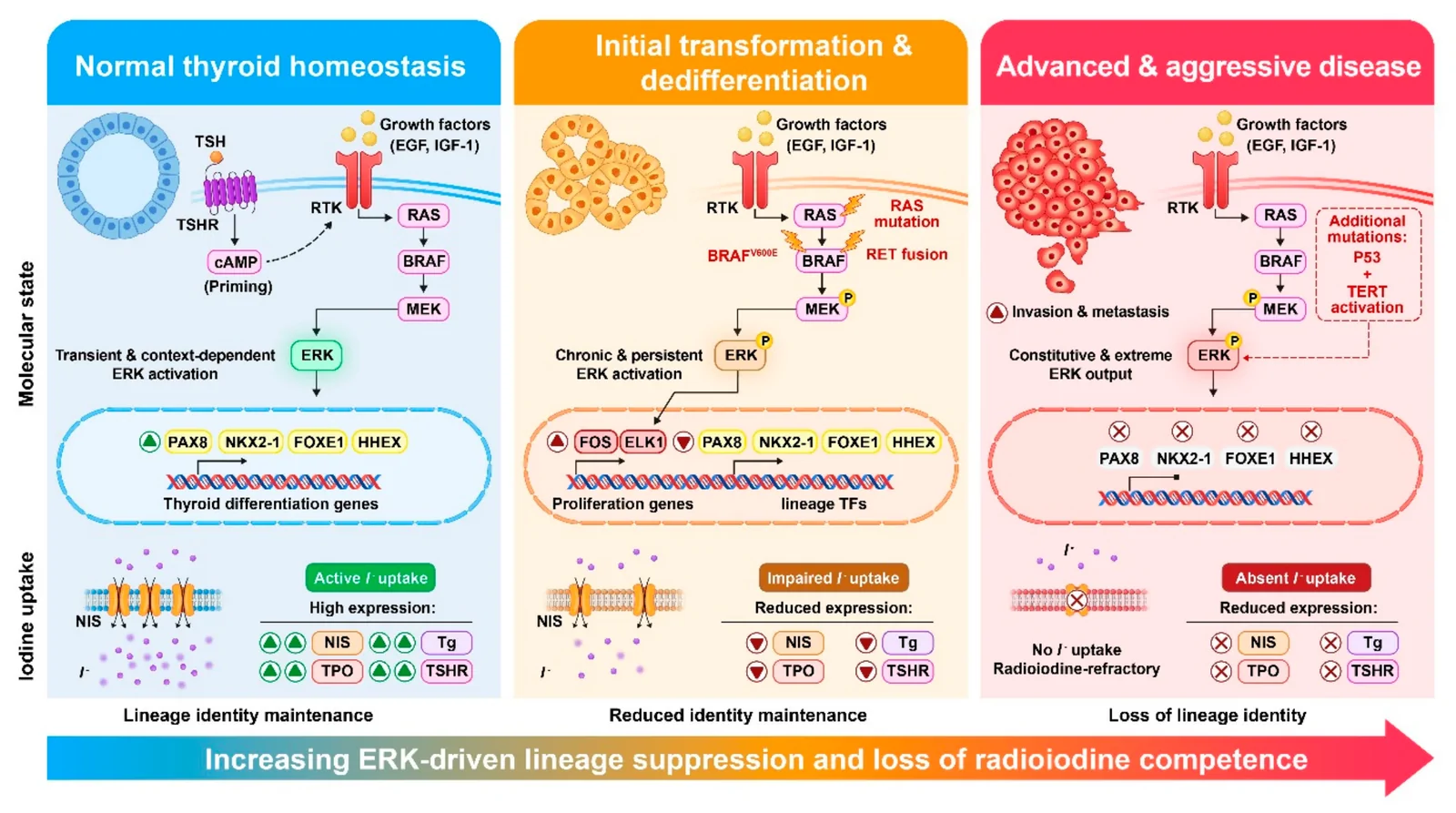
Progressive ERK-driven suppression of thyroid lineage identity and radioiodine competence. Schematic representation of the transition from normal thyroid homeostasis to oncogenic transformation and advanced disease. In normal follicular cells, *TSH/TSHR–cAMP* signaling and regulated receptor tyrosine kinase input produce transient, context-dependent ERK activation while maintaining lineage transcription factors, including *PAX8, NKX2-1, FOXE1*, and *HHEX*, and preserving *NIS*-mediated iodide uptake and expression of thyroid-specific genes. During transformation, *MAPK*-activating alterations such as *BRAF^V600E*, *RAS* mutations, and *RET* fusions sustain *ERK* signaling, promote proliferative transcriptional programs, and progressively suppress thyroid-lineage factors and iodine-handling genes, including *NIS*, *TPO, TG*, and *TSHR*. In advanced disease, additional alterations such as *TP53* loss and *TERT* activation accompany intensified *ERK* output, invasion and metastasis, further loss of lineage identity, and absent or markedly impaired iodide uptake, culminating in a RAIR phenotype.

At the aggressive end of the spectrum, progression from DTC to PDTC and anaplastic TC usually preserves the founding *MAPK* driver. Still, it acquires additional alterations that deepen the collapse of thyroid identity. Genomic and transcriptomic analyses of PDTC and ATC showed frequent secondary tumor-suppressor events, with TP53 mutations markedly enriched in ATC relative to PDTC. At the same time, more recent experimental work demonstrated that TERT reactivation accelerates *BRAF*-driven TC dedifferentiation and progression [35,36]. These data support a coherent model for *ERK* biology in TC: *MAPK* pathway activation establishes the initial lineage-biased tumor state, whereas cooperating alterations determine how far that state progresses toward high-grade, RAIR disease (Figure 1) [35,36,42].

## 4. ERK-Driven Nuclear Transcriptional Reprogramming in Thyroid Cancer

In TC, especially in *BRAFV600E*-driven papillary tumors, *ERK* signaling is not only a proliferative pathway but also a determinant of cell identity [54,55]. Review-level synthesis and genomic profiling converge on the conclusion that the magnitude of MAPK/ERK flux is a major determinant of differentiation state, invasive phenotype, and responsiveness to redifferentiation therapy [56]. In the TCGA PTC cohort, *ERK* output and thyroid differentiation were inversely coupled, with *BRAF*-like tumors showing stronger *MAPK* transcriptional output and lower expression of thyroid functional genes [16,42].

A particularly instructive demonstration of *ERK*’s dual transcriptional output came from the inducible *BRAFV600E* mouse thyroid model [37]. In that system, oncogenic BRAF rapidly increased canonical *MAPK* transcriptional-output genes such as *DUSP5* and *PLAT*, while simultaneously causing near-complete loss of *NIS, Tg, TPO,* and *TSHR*, together with profound repression of the lineage transcription factors *Pax8* and *Ttf-2* and a lesser decrease in *Ttf-1* [37]. When *BRAFV600E* expression was extinguished, thyroid follicular architecture, thyroid-specific gene expression, and RAI incorporation were restored, establishing that *ERK*-driven activation of oncogenic output and *ERK*-driven repression of differentiation are mechanistically linked and, at least in part, reversible [37].

This duality is also reflected in human disease and pharmacological studies [16]. The TCGA framework showed that tumors with higher *ERK* scores have lower TDS, and a JCI study further demonstrated that even a modest increment in *ERK* suppression markedly increased thyroid differentiation gene expression and iodine accumulation in *BRAFV600E* TCs [16,52]. Taken together, these data support a model in which *ERK* does not simply “turn on” growth-related transcription; rather, it rewires the nuclear program toward a coupled state of oncogenic activation and lineage extinction.

At the nuclear level, *ERK* classically signals through *ETS*-domain transcription factors such as ELK1, which directly regulates immediate-early genes, including *FOS* and *EGR1* [57]. In TC, this early-response logic is then stabilized into tumor-promoting transcriptional circuits. One of the clearest examples is the *BRAFV600E/MAPK–FOS–GABP* axis: the pathway phosphorylates and activates *FOS, FOS* binds and activates the *GABPB* promoter, and the resulting *GABPA–GABPB* complex selectively activates the mutant *TERT* promoter [58,59]. This mechanism provides a direct molecular link between *ERK* signaling and the aggressive biology of TCs harboring both *BRAFV600E* and *TERT*-promoter alterations.

*ERK*-dependent induction of oncogenic transcription factors in TC is not limited to telomerase regulation. In PTC cells, *ETV5* is expressed downstream of the *MAPK* pathway, is required for tumor-cell growth, and directly upregulates *TWIST1*, connecting *ERK* activity to epithelial–mesenchymal and metastatic transcriptional programs [60]. Thus, a central nuclear consequence of *ERK* activation in TC is the installation of a transcription factor hierarchy that converts transient mitogenic signaling into sustained programs of proliferation, invasion, and progression [48,61].

The thyroid differentiation program is maintained by a lineage-defining transcriptional network centered on *NKX2-1, PAX8, FOXE1*, and *HHEX* [22,23]. These factors are co-expressed during thyroid specification and remain fundamental for the maintenance of differentiated thyrocyte identity [62]. In TC, *ERK* activation antagonizes this network directly and functionally. In the inducible BRAFV600E model, *ERK* activation profoundly reduced *PAX8, TTF-2/FOXE1*, and *TTF-1/NKX2-1* together with the major iodine-handling genes, showing that lineage-factor loss is an intrinsic part of the *ERK* output rather than a late by-product of tumor progression [23,37].

Mechanistically, one of the best-established routes for this antagonism is the *BRAFV600E–TGF-β–SMAD* axis [63]. *BRAFV600E* induces *TGF-β* secretion, and the resulting pathway represses *NIS* and promotes a more malignant phenotype. At the transcription-factor level, *Smad3* physically and functionally interacts with *PAX8*, decreasing *PAX8* DNA binding as well as *PAX8* mRNA and protein levels, thereby impairing *PAX8*-dependent transactivation of thyroid-specific genes [31]. More recently, a distinct but conceptually aligned mechanism was described in which *MAPK* signaling upregulates *USP15* to stabilize the developmental transcription factor *TBX3*, thereby reactivating a developmental, differentiation-restricting state that supports *BRAF/MAPK*-mediated dedifferentiation and tumorigenesis [64]. Collectively, these data indicate that *ERK* suppresses thyroid lineage identity both by directly weakening lineage-transcription-factor function and by reactivating developmental repressors that oppose terminal differentiation.

The available TC data indicate that *ERK*-dependent repression is executed not only through altered transcription-factor abundance and activity but also through chromatin-based co-repressor mechanisms. The strongest promoter-level evidence concerns *NIS*: *BRAFV600E*-promoted *NIS* silencing is associated with histone deacetylation at the *NIS* promoter, and restoration of *MAPK* signaling blockade increases promoter acetylation [65]. Functionally, simultaneous suppression of *BRAFV600E* and histone deacetylase activity robustly re-induced thyroid-gene expression and RAI uptake, while follow-up work showed that *MAPK* inhibitors further enhance *HDAC* inhibitor-induced redifferentiation in *BRAFV600E* PTC cells [66]. These findings support *HDAC*-dependent chromatin repression as a major execution arm of *ERK*-driven dedifferentiation.

A second repressive axis involves *EZH2/PRC2*-associated *H3K27* trimethylation. Evidence from human thyroid tissues indicates that *EZH2* overexpression is most clearly associated with undifferentiated ATC, whereas well-differentiated papillary and follicular carcinomas did not show comparable generalized *EZH2* overexpression [67]. In *BRAF^V600E*-mutant PTC cell-line models, pharmacologic *EZH2* inhibition enhanced *MAPK* inhibitor-induced expression of differentiation-associated genes and decreased global *H3K27* trimethylation [68]. These observations support a potential role for *PRC2/EZH2*-mediated repression in selected experimental contexts but do not establish constitutive *EZH2* hyperactivity as a universal feature of *BRAFV600E*-positive human PTC [31,65,68,69]. Notably, the promoter-resolved evidence is strongest for *NIS* and *PAX8*, whereas the full map of *ERK*-dependent co-repressor occupancy across the broader thyroid lineage network remains less completely defined [70].

**Figure 2:**
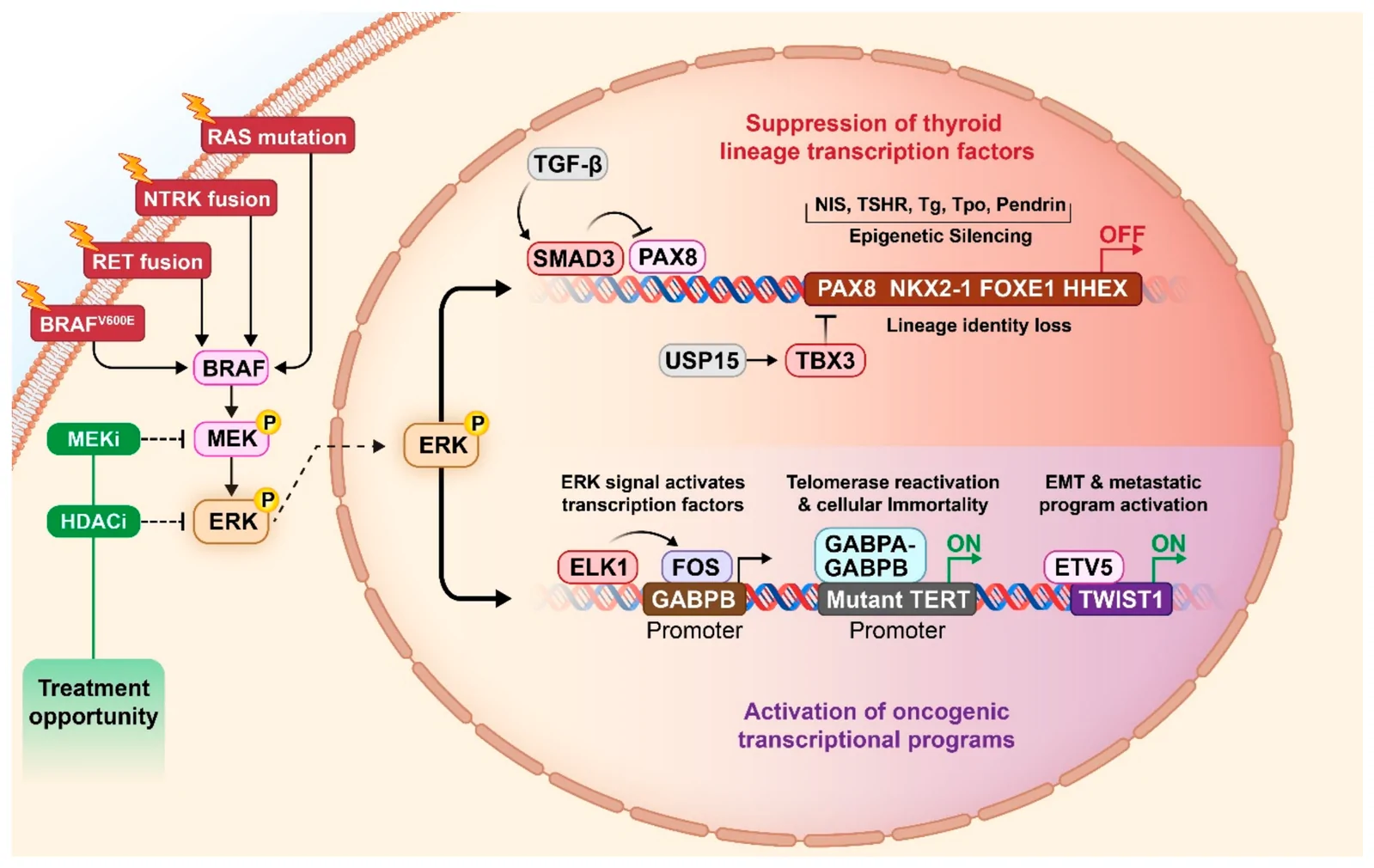
Nuclear transcriptional consequences of oncogenic ERK activation in thyroid cancer. *MAPK*-activating alterations, including *BRAF^V600E*, *RAS* mutations, and *RET/NTRK* fusions, converge on sustained *MEK*–*ERK* signaling and generate two complementary nuclear outputs. ERK-associated *TGF-β/SMAD3–PAX8* signaling and *USP15–TBX3* activity suppress thyroid-lineage transcription factors and iodine-handling genes, promoting lineage identity loss and epigenetic silencing. In parallel, ERK activates oncogenic transcriptional programs through *ELK1–FOS–GABP*, facilitating mutant *TERT* promoter activation and cellular immortality, and through *ETV5–TWIST1*, promoting epithelial–mesenchymal and metastatic programs. *MEK* and *HDAC* inhibition represent potential points of therapeutic intervention aimed at attenuating oncogenic *ERK* output and restoring thyroid differentiation.

The mechanistic literature remains strongest for *BRAFV600E*-driven PTC and genetically engineered mouse models, whereas the corresponding nuclear circuitry is less completely resolved in *RAS*-like tumors and in the later stages of poorly differentiated and anaplastic progression. Likewise, the existence of *HDAC*- and *EZH2*-dependent repression is well supported, but promoter-by-promoter mapping of the exact *ERK*-linked co-repressor assemblies across the full thyroid lineage-transcription network is still comparatively limited (Figure 2).

## 5. Epigenetic Enforcement of Functional Dedifferentiation

The hyperactivation of the *MAPK/ERK* signaling cascade, frequently driven by *BRAF V600E* or *RAS* mutations, acts as a primary engine of TC dedifferentiation [71]. Beyond immediate transcriptional changes, *ERK* hyperactivation can reinforce long-term dedifferentiation through epigenetic reprogramming, contributing to persistent impairment of RAI responsiveness and aggressive phenotypic features [18,72]. These epigenetic mechanisms—spanning histone modifications, DNA methylation, and non-coding RNA networks—act synergistically to silence essential thyroid-specific genes such as *NIS* (*SLC5A5*), *TSHR*, and *TTF-1*.

**Figure 3:**
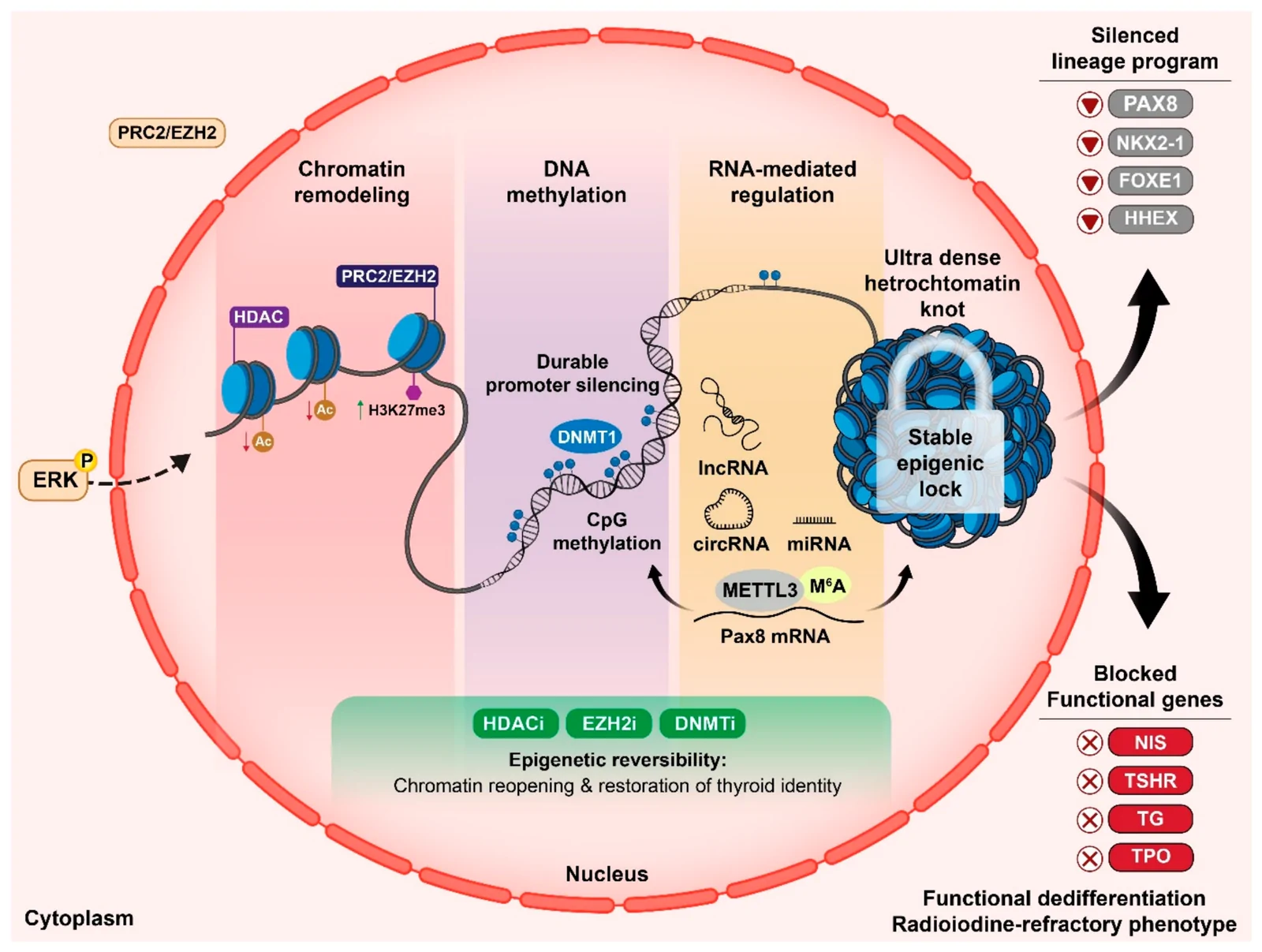
Epigenetic enforcement of ERK-driven functional dedifferentiation in thyroid cancer. Sustained *ERK* signaling promotes a repressive nuclear state through coordinated chromatin remodeling, DNA methylation, and RNA-mediated regulation. HDAC-dependent histone deacetylation and *PRC2/EZH2*-associated *H3K27* trimethylation reduce chromatin accessibility, while *DNMT1*-mediated CpG methylation contributes to durable promoter silencing. In parallel, lncRNAs, circRNAs, miRNAs, and *METTL3*-mediated m^6A modification modulate lineage-associated transcripts and reinforce epigenetic repression. These mechanisms converge on silencing of thyroid-lineage transcription factors, including *PAX8, NKX2-1, FOXE1*, and *HHEX*, and suppression of functional genes such as *NIS, TSHR, TG*, and *TPO*, thereby stabilizing a RAIR phenotype. Pharmacologic targeting of *HDAC*, *EZH2*, or *DNMT* illustrates potential strategies to reopen chromatin and restore thyroid-lineage identity.

Histone modifications, particularly acetylation and methylation, dynamically govern chromatin accessibility and dictate the expression of key thyroid differentiation markers [73,74]. The oncogenic activation of the *MAPK* pathway actively disrupts this epigenetic balance. For instance, the *BRAF* V600E mutation maintains histones at critical regulatory regions of the *NIS* promoter in a deacetylated, transcriptionally repressive state [65]. Consequently, treatment with histone deacetylase inhibitors (e.g., *SAHA*) or *MEK* inhibitors increases histone acetylation at the *NIS* promoter, reactivating its expression and providing a molecular basis for redifferentiation therapies in RAIR disease (Figure 3) [65].

Beyond acetylation, specific chromatin remodeling is heavily influenced by histone methylation dynamics [74]. Epigenetic regulators orchestrate the trimethylation of *H3K36* at promoters of chromatin remodelers like *SMARCA2*, which subsequently bind to enhancers of critical thyroid transcription factors (e.g., *PAX8* and *FOXE1*) to promote chromatin accessibility [74]. Similarly, profound losses of histone acetylation characterize the suppression of both the *TTF-1* (*NKX2-1*) promoter and the *RARβ2* promoter, the latter of which drives resistance to retinoic acid therapies unless treated with broad-spectrum *HDAC* inhibitors [73,75,76].

Aberrant DNA methylation, predominantly hypermethylation of CpG islands in gene promoters, acts as a durable silencer of thyroid-specific function [77]. Unlike the highly dynamic nature of histone acetylation, DNA methylation often represents a more stable and long-term enforcement of the dedifferentiated phenotype. The *TSHR* gene promoter is frequently hypermethylated in papillary and follicular TCs, leading directly to TSH unresponsiveness and gene silencing, whereas it remains completely unmethylated in normal tissues [78]. Similarly, the *NIS* promoter is heavily targeted by DNA methylation, a process interconnected with DNA methyltransferase 1 (DNMT1) upregulation and upstream oncogenic kinase cascades [79,80].

Epigenetic plasticity is also highly evident during tumor invasion and metastasis. Promoters of epithelial markers, such as *CDH1* (*E-cadherin*), often undergo hypermethylation in primary tumors to facilitate epithelial-to-mesenchymal transition (EMT), whereas they may revert to unmethylated states in lymph node metastases to enable colonization [80,81]. Conversely, dedifferentiation also exploits pathological hypomethylation. For example, tumor suppressor and regulatory genes like *Maspin* paradoxically undergo promoter hypomethylation in advanced, undifferentiated TCs, driving aberrant overexpression that correlates with severe morphological dedifferentiation [82].

Non-coding RNAs (ncRNAs)—encompassing microRNAs (miRNAs), circular RNAs (circRNAs), and long non-coding RNAs (lncRNAs)—constitute an intricate post-transcriptional and epigenetic regulatory web governing thyroid dedifferentiation [83,84]. The oncogenic activation of the *MAPK/ERK* pathway triggers vast deregulation of miRNAs that target essential iodine metabolism genes, thereby amplifying tumor aggressiveness by modulating the dual function of *TGFβ*; this switches *TGFβ* from an antimitogenic factor in normal thyrocytes to a potent inducer of EMT and metastasis in cancer cells [72].

Circular RNAs frequently function as competing endogenous RNAs (ceRNAs) to sponge these miRNAs and relieve target gene suppression. For example, *Hsa_circ_0023990* is significantly upregulated in dedifferentiated TC tissues; it sponges miR-485-5p to upregulate *FOXM1*, thereby accelerating tumor proliferation and glycolysis [85,86]. Furthermore, this specific circRNA network has been implicated in exacerbating iodine uptake disorders by modulating *NIS* promoter methylation through a highly integrated miR-448/DNMT1 signaling axis. Similarly, *CircRTN1* acts as a cancer-promoting molecule in TC by sponging miR-431-5p, which in turn upregulates *TGFA* and accelerates disease progression [87].

Beyond the ceRNA networks, RNA methylation—specifically N6-methyladenosine (m6A)—has emerged as a critical epigenetic dimension. RNA m^6A modification may also influence thyroid differentiation through chromatin-regulatory networks. METTL3-mediated m^6A modification promotes SETMAR expression in an IGF2BP3-dependent manner; SETMAR subsequently enhances SMARCA2 transcription, facilitating chromatin accessibility at PAX8- and FOXE1-associated regulatory regions [74,88]. Together with the broader exosomal ncRNA microenvironment—which dynamically modulates proliferation, EMT, angiogenesis, and immunoediting—these RNA-level epigenetic modifications establish a comprehensive molecular map of TC dedifferentiation, offering promising targets for future redifferentiation therapies [88].

## 6. Experimental and Preclinical Evidence Supporting ERK-Driven Dedifferentiation

Experimental and preclinical evidence support *ERK/MAPK* signaling as a major regulator of TC dedifferentiation, particularly in follicular-cell-derived TC models, including PTC, FTC, PDTC, and ATC [49,52,71]. Across cell-line systems, genetically engineered mouse models, organoid-based models, zebrafish models, and patient-derived molecular datasets, activation of the *RAF–MEK–ERK* axis was consistently associated with reduced thyroid-specific differentiation programs, whereas pharmacologic inhibition of *MEK/ERK* signaling restored differentiation markers and, in selected models, functional iodide uptake [49,50,52,71,72,89].

Cell-line studies provide direct experimental evidence that *ERK* pathway activation suppresses thyroid differentiation gene expression in TC models [50,52,72,90,91]. In *PCCL3* thyroid cells, inducible expression of *RET/PTC3* inhibited TSH-induced thyroglobulin and *NIS* expression, and this dedifferentiating effect was mediated through Y1062-dependent *SHC–RAS–MAPK* signaling [49]. *RET/PTC* constructs that activated *SHC–RAS* signaling induced loss of differentiation markers, whereas a construct unable to bind SHC did not produce the same dedifferentiating phenotype, supporting a specific role for the *RAS*–*MAPK/ERK* branch rather than nonspecific *RET/PTC* signaling [49,92]. Acute expression of oncogenic *H-RAS*(V12) or constitutively active *MEK1* similarly suppressed TSH-induced thyroglobulin and *NIS* expression, placing *MEK/ERK* activation downstream of oncogenic *RAS* signaling in the repression of thyroid differentiation genes [49]. Treatment with *MEK* inhibitors restored thyroglobulin and *NIS* expression in this model, demonstrating that *ERK*-driven dedifferentiation is at least partially reversible after pathway inhibition [93,94].

Additional TC cell-line evidence supports the relationship between *MAPK/ERK* signaling and dedifferentiated phenotypes [90,91,95]. Comprehensive molecular characterization of authenticated human TC cell lines showed that these models are profoundly dedifferentiated, supporting their use for studying molecular events associated with loss of differentiated thyroid identity [90]. In poorly differentiated TC cells, *EGFR* inhibition with erlotinib inhibited *EGF*-induced *ERK1/2* signaling and enhanced the activity of cytotoxic agents, linking growth-factor-driven *ERK* activation to aggressive, poorly differentiated TC biology [91]. In undifferentiated human TC cells, *DDR1* silencing increased thyroid differentiation markers, including *NIS*, *Tg, TSHR*, and *TPO*, while reducing EMT and stemness-associated markers such as *vimentin, Snail-2, Zeb1, Zeb2, N-cadherin, OCT-4, SOX-2, ABCG2*, and *Nanog* [91,95]. Although the *DDR1* study focused on *IGF-2/IR-A* signaling, its findings support the broader concept that signaling networks connected to *ERK*-associated oncogenic programs regulate differentiation, EMT, and stemness phenotypes in aggressive TC cells.

Genetically engineered mouse models provide strong in vivo evidence that oncogenic *ERK* pathway activation promotes TC dedifferentiation [50,52,72,96]. Targeted thyroid-specific expression of *BRAFV600E* in transgenic mice induced PTCs with aggressive histologic features, including tall-cell morphology, local invasion, and foci of poorly differentiated carcinoma [50]. These *BRAFV600E*-driven tumors showed reduced expression of thyroid differentiation markers, including *TPO, NIS*, and *Tg*, demonstrating that constitutive activation of the *MAPK/ERK* pathway is sufficient to drive both tumorigenesis and loss of differentiated thyroid function in vivo [46,50,72].

In *TPO-Cre LSL-BRAFV600E* mice and *BRAF*-mutant thyroid cell models, *BRAF*-driven *ERK* activation was associated with suppression of genes required for iodide transport and thyroid differentiation, including *NIS, TPO, Tg*, and *TSHR* [52]. Pharmacologic inhibition of *MEK* restored expression of thyroid differentiation genes and improved RAI responsiveness, showing that *ERK*-dependent repression of the iodide-handling program is therapeutically reversible [52,97]. Importantly, sustained and profound *ERK* inhibition produced stronger restoration of *NIS* expression and RAI response than transient or incomplete *ERK* inhibition, indicating that the depth and duration of *ERK* pathway suppression are critical determinants of redifferentiation [52].

Other in vivo models further support *ERK* signaling as part of a broader oncogenic network controlling dedifferentiation [50,71,72,96]. In *Dicer1*-deficient and *BRAFV600E*-related models, activation of the *MAPK*/*ERK* pathway was associated with downregulation of thyroid differentiation markers, including *NIS*, *TPO*, and *Tg*, along with EMT-associated changes such as increased *vimentin* and reduced *E-cadherin* [46,61,72]. This study also identified *ETV5* and *TWIST1* as transcriptional mediators linking *MAPK*/*ERK* activation to EMT and loss of differentiation in thyroid cells [60]. In murine ATC models and mESC-derived TC organoids, oncogenic *BRAF*-associated *MAPK* activation suppressed thyroid-lineage genes, including *Tg, NIS/SLC5A5, TPO, TSHR, PAX8, SLC26A4, DIO1,* and *DUOX2* [71]. In these ATC-related systems, *BRAF* and *MEK* inhibition restored differentiation-associated gene expression and improved iodine uptake in selected contexts, supporting the reversibility of *MAPK/ERK*-driven dedifferentiation even in highly aggressive TC models [71].

ERK-driven dedifferentiation is modulated by pathway crosstalk, particularly with *PI3K/AKT* signaling [61,71,98]. In a thyroid epithelium model combining oncogenic *Kras* activation with *Pten* deletion, simultaneous *PI3K* activation was required to sustain *ERK* activation and transform thyroid epithelial cells in vivo [96]. *PI3K* activation suppressed *Kras*-induced negative feedback mechanisms that would otherwise uncouple *MEK* from *ERK* activation, thereby maintaining *ERK* signaling in transformed thyroid cells [96]. Combined pharmacologic inhibition of *PI3K* and *MAPK* signaling completely inhibited the growth of double-mutant TC cell lines, indicating functional cooperation between these pathways in maintaining malignant TC phenotypes [10,96,99]. In ATC-related models, combined *MAPK* and *PI3K* pathway alterations similarly contributed to dedifferentiation and therapeutic resistance, supporting the concept that *ERK* signaling acts as a central but context-dependent driver within a broader oncogenic signaling network [71].

Additional molecular interactions also influence ERK-associated dedifferentiation phenotypes [72,89,90]. Loss of *p53* function was identified as a prominent event in human TC cell-line immortalization and may contribute to the dedifferentiated state of these models [90]. *MAPK/ERK* activation also interacted with *TGFβ*-related signaling and microRNA deregulation in thyroid cells, linking *ERK* pathway activation to transcriptional and post-transcriptional mechanisms that promote loss of differentiation and EMT [72]. In patient-derived advanced TC samples, alterations in *SWI/SNF* chromatin remodeling genes and *PI3K–AKT–mTOR, MAPK*, and *JAK–STAT* pathways increased across more dedifferentiated histologies, suggesting that *ERK* pathway activation cooperates with chromatin and signaling alterations during TC progression [89].

Patient-sample data provide translational support for the experimental findings observed in preclinical models [89]. In a cohort of advanced, recurrent, and/or metastatic thyroid carcinomas analyzed by targeted next-generation sequencing, whole-exome sequencing, and RNA profiling, genomic alterations involving *MAPK* signaling were enriched across more dedifferentiated disease states [89]. Among tumors that dedifferentiated after *BRAF* inhibitor therapy, most harbored alterations in pathways including *MAPK, PI3K–AKT–mTOR, JAK–STAT*, or *SWI*/*SNF* chromatin remodeling, supporting the clinical relevance of pathway cooperation during progression from differentiated to dedifferentiated TC [71,89,100,101]. *ERK* signaling activation was also observed in a vemurafenib-treated tumor after anaplastic transformation, linking *MAPK* pathway reactivation to clinical dedifferentiation after targeted therapy exposure [89].

## 7. Theranostic Implications: Redifferentiation as a Targetable State

RAIR differentiated TC represents a clinically important state in which follicular-lineage tumors retain malignant growth capacity but lose sufficient thyroid differentiation to concentrate iodine effectively. The therapeutic rationale for redifferentiation is therefore not simply cytoreduction, but pharmacologic restoration of a targetable thyroid phenotype, principally sodium–iodide symporter-mediated iodine transport, followed by lesion-directed or whole-body therapeutic RAI. This concept is strongly supported by mechanistic models showing that oncogenic *MAPK* activation suppresses thyroid-lineage genes and RAI incorporation, and that pharmacologic *MAPK* inhibition can restore iodine uptake in *BRAF*-driven TC models [37,52].

The most mature redifferentiation strategy in RAIR-TC is inhibition of the *RAF–MEK–ERK* axis [97]. This approach arose from preclinical work showing that conditional *BRAF* activation in thyroid cells suppresses thyroid-specific gene expression and RAI incorporation, whereas small-molecule *MAPK* inhibitors restore RAI uptake in vivo [37]. Genomic analyses of PTC further support this biological framework: TCGA showed that driver classes are associated with distinct signaling and differentiation states, and that *BRAF*-like tumors generally display lower TDS than *RAS*-like tumors [16].

Clinical proof of concept was provided by Ho et al., in which selumetinib increased iodine-124 uptake in 12 of 20 patients with advanced RAIR-TC; 8 patients reached the dosimetry threshold for iodine-131 therapy, and among treated patients,5 had confirmed partial responses and 3 had stable disease [38]. The response was particularly notable in *NRAS*-mutant tumors, in which all 5 patients showed increased iodine uptake sufficient for treatment. Subsequent prospective and genotype-guided studies extended this principle using *MEK* inhibition for *BRAF*-wild-type or *RAS*-mutant tumors and combined *BRAF/MEK* inhibition for *BRAFV600E*-mutant tumors. The ERRITI study used trametinib in *BRAF*-wild-type disease and dabrafenib plus trametinib in *BRAF*-mutant disease, with RAI re-treatment guided by restored uptake on imaging [102]. The MERAIODE phase II program similarly evaluated trametinib plus RAI in metastatic *RAS*-mutated RAI-differentiated TC, and a parallel *BRAFV600E* cohort evaluated dabrafenib plus trametinib followed by RAI [103,104].

*BRAF*-selective inhibition has also been explored as a redifferentiation approach. In a pilot trial of vemurafenib in *BRAF*-mutant RAIR-TC, patients underwent iodine-124 PET before and after approximately 4 weeks of therapy, and those exceeding a lesional dosimetry threshold proceeded to iodine-131 treatment [105]. Dabrafenib-based redifferentiation has likewise been reported in *BRAFV600E*-mutant metastatic PTC, supporting the broader concept that *ERK*-pathway suppression can restore iodine avidity in a subset of *BRAF*-driven tumors [106].

Serum Tg and *NIS* expression should not be interpreted as interchangeable measures of differentiation during kinase therapy. Declining circulating Tg may reflect reduced viable tumor burden, altered secretion, or treatment-associated changes in tumor biology; in the DECISION trial, sorafenib produced substantial Tg reductions in many patients, consistent with its use as a pharmacodynamic marker of treatment response rather than a direct measure of lineage restoration [107]. Conversely, *NIS* restoration is a cell-intrinsic functional process that requires transcription, membrane trafficking, and the preservation of iodide transport. Enhanced *NIS* function is not a general class effect of multikinase inhibitors: for example, experimental lenvatinib effects on iodine uptake were highly model-dependent, and pretreatment even reduced tumor iodine uptake in vivo in an *NIS*-expressing xenograft model [108]. Thus, a reduction in serum Tg does not conflict mechanistically with increased *NIS* activity in selected experimental settings.

Combination strategies are being pursued because *MAPK* inhibition alone does not uniformly restore clinically meaningful RAI uptake. One rationale is that *ERK* signaling is not the only determinant of the differentiated thyroid phenotype; parallel survival pathways, adaptive receptor tyrosine kinase signaling, and epigenetic repression may all constrain *NIS* re-expression and membrane localization. In *BRAF*-mutated TC organoid models, dual inhibition of *MAPK* and *PI3K* pathways reversed *BRAF*-driven dedifferentiation and restored follicular organization and thyroid function more effectively than single-pathway targeting, suggesting that *PI3K* signaling can cooperate with *MAPK* signaling in maintaining a dedifferentiated state [103,109,110].

Adaptive *MAPK* rebound is another major rationale for combination therapy. Cheng et al. showed that *HER2*/*HER3* activation can mediate resistance to *BRAF*/*MEK* inhibitor-induced redifferentiation in *BRAFV600E* PTC cells, and that adding the *HER* inhibitor lapatinib prevented *MAPK* rebound and enhanced iodine-handling gene expression and RAI uptake [111]. Epigenetic co-targeting is also biologically plausible. Fu et al. reported that panobinostat increased iodine-metabolizing gene expression and RAI uptake, and that combining panobinostat with dabrafenib or selumetinib produced a stronger *BRAF^V600E*-dependent redifferentiation effect through increased histone acetylation at the *NIS* promoter [69]. These findings are consistent with earlier data indicating that *BRAF^V600E* can regulate *NIS* expression through epigenetic mechanisms, including *DNMT1*–linked repression [112].

Earlier redifferentiation approaches using retinoids produced variable and generally modest results, but they remain historically important because they established the clinical objective of restoring iodine avidity before targeted kinase therapy became available. Retrospective data examining 13-cis-retinoic acid with RAI in RAIR-PTC suggested heterogeneous outcomes and raised the possibility that mutational context may influence response [113]. Current combination strategies have therefore shifted from empiric redifferentiation toward biologically matched regimens: *MAPK* inhibition as the backbone, with *PI3K*, *HER*-family, epigenetic, or lineage-directed agents added according to resistance biology and molecular context.

Redifferentiation therapy is intrinsically theranostic because the same biological process—restored iodine handling—is first measured diagnostically and then exploited therapeutically. Ho et al. used iodine-124 PET/CT before and after selumetinib to quantify lesional iodine uptake and determine whether patients met a dosimetry threshold for iodine-131 therapy [38]. Vemurafenib redifferentiation studies similarly used iodine-124 PET to identify patients in whom BRAF inhibition increased lesional RAI concentration sufficiently to justify therapeutic iodine-131 [105]. These trial designs emphasize that redifferentiation should not be defined only by molecular pathway inhibition, but by demonstrable restoration of iodine uptake at a level likely to deliver a meaningful absorbed radiation dose.

Personalization also depends on tumor genotype. The International Thyroid Oncology Group statement (ITOG) summarized that redifferentiation has been evaluated in *BRAF*-mutated and *RAS*-mutated tumors in prospective trials, and in selected tumors with *RET* or *NTRK* fusions; across studies, iodine uptake restoration ranged from 33% to 95%, and tumor response rates ranged from 11% to 80%, with substantial heterogeneity in eligibility criteria, imaging methods, treatments, and endpoints [114]. These data support a precision framework in which molecular testing identifies the dominant driver alteration, *MAPK*-pathway inhibition is selected accordingly, and post-treatment iodine imaging determines whether RAI therapy is justified. In this framework, *RAS*-mutant or *BRAF*-wild-type disease may be approached with *MEK* inhibition, whereas *BRAF^V600E*-mutant disease may require *BRAF* inhibition alone or combined *BRAF/MEK* inhibition to achieve sufficient *ERK* suppression.

Functional imaging adds another layer of personalization. FDG-avid RAI-negative lesions often represent less differentiated disease, while restoration of iodine uptake after MAPK inhibition can identify lesions that have reacquired a therapeutically exploitable phenotype. Iodine-124 PET/CT and lesion-level dosimetry are particularly important because restored uptake is often heterogeneous across metastases; a patient may have some lesions that redifferentiate and others that remain iodine-refractory [38,105]. Thus, the most rigorous application of redifferentiation therapy should integrate genotype, lesion-level iodine imaging, tumor kinetics, FDG-PET phenotype, and individualized dosimetry rather than relying solely on a fixed drug-exposure interval or a diagnostic whole-body scan.

*NIS* immunohistochemistry (IHC) is biologically attractive as a potential marker of RAI competence but is not currently sufficiently validated for routine treatment selection. Total *NIS* protein abundance does not necessarily indicate basolateral membrane localization or functional iodide transport, and *NIS* expression in a resected primary tumor may not reproduce the phenotype of recurrent or metastatic lesions [70,115,116]. Contemporary molecular/IHC analyses support an association between thyroid-lineage marker expression and iodine avidity, but predictive performance remains insufficient to replace direct functional assessment [115]. Accordingly, routine *NIS* IHC cannot presently be recommended as a therapeutic index before RAI; standardized membrane-specific quantitative *NIS* assessment remains a candidate biomarker for prospective validation [115,116,117].

Despite strong biological rationale and encouraging early clinical activity, redifferentiation therapy remains limited by heterogeneous response, incomplete durability data, and uncertain incremental benefit over kinase inhibition alone in some contexts. Published trials vary substantially in genotype selection, drug regimen, treatment duration, imaging modality, dosimetry threshold, RAI activity, prior therapies, and response endpoints. The ITOG statement highlighted wide variability in iodine uptake restoration and tumor response rates across trials, underscoring that redifferentiation is not yet a standardized intervention [114]. In addition, the phase III ASTRA trial tested selumetinib plus adjuvant RAI in high-risk differentiated TC and did not improve outcomes, indicating that *MAPK* inhibition cannot be assumed to improve RAI efficacy outside carefully selected RAIR redifferentiation settings [118].

Resistance can occur at several biological levels. First, *MAPK* suppression may be insufficient or transient; sustained *ERK* inhibition was shown to maximize RAI response in *BRAF^V600E* TC models, indicating that the depth and duration of pathway blockade are critical determinants of redifferentiation [52]. Second, adaptive feedback can reactivate *ERK* signaling despite upstream inhibition, as demonstrated by *HER2/HER3*-mediated *MAPK* rebound after *BRAF/MEK* inhibition [69]. Third, parallel pathways such as *PI3K* may maintain dedifferentiation, invasion, or survival even when *MAPK* is inhibited; organoid studies support combined *MAPK* and *PI3K* targeting as a strategy to overcome this biology [103,104]. Fourth, epigenetic repression of iodine-handling genes may persist after kinase inhibition, explaining why *HDAC* inhibition can enhance *MAPK* inhibitor-induced *NIS* re-expression in *BRAF^V600E* models [69,112]. The principal prospective clinical studies evaluating pharmacologic restoration or enhancement of radioiodine avidity in differentiated thyroid cancer, together with their efficacy outcomes, toxicity profiles, and key interpretive limitations, are summarized in Table 1.

Collectively, these studies establish biological proof that *MAPK*-pathway inhibition can restore iodine avidity in a subset of RAIR tumors, but they do not establish uniformly effective clinical redifferentiation. Cross-trial comparisons are limited by small cohorts, differing molecular-selection strategies, definitions of RAI refractoriness, duration and depth of pathway inhibition, imaging methods, dosimetry criteria, ^131I activities, and response endpoints [114]. Importantly, restored iodine uptake is an intermediate biological endpoint rather than a validated surrogate for therapeutic benefit: increased avidity does not necessarily ensure adequate retention, absorbed dose, or durable structural response. A 2026 meta-analysis estimated a pooled restoration rate of approximately 50% and an objective response rate of approximately 29%, underscoring the substantial proportion of patients who do not obtain meaningful clinical benefit [124]. Accordingly, redifferentiation should presently be considered a biologically supported but incompletely validated strategy rather than an established reversal of RAIR disease [114,117,124]. This cautious interpretation is consistent with the 2025 ATA guidelines, which state that *MAPK*-blockade redifferentiation may be considered in selected patients with progressive RAIR-DTC harboring targetable alterations, while encouraging clinical-trial participation; the recommendation is conditional and based on low-certainty evidence. In contrast, redifferentiation is not recommended as an adjuvant strategy in unselected high-risk DTC [117].

## 8. Challenges and Future Perspectives

### 8.1. Biomarker-Defined Patient Selection and Metastatic Heterogeneity

The next generation of redifferentiation studies should move beyond single-driver selection toward composite measures of reversible thyroid-lineage suppression. Candidate biomarkers include driver genotype, quantitative *MAPK/ERK* output, TDS, residual iodine-handling gene expression, FDG phenotype, epigenetic state, and markers of parallel signaling activation [16,17,52,89,105,114]. Importantly, whether TDS or *MAPK*-output scores measured in the primary tumor can predict redifferentiation of synchronous or metachronous metastases remains unknown. Metastatic lesions may acquire additional genomic, transcriptional, and epigenetic alterations during progression and therapy, and existing redifferentiation studies incorporating molecular profiling have generally evaluated contemporary metastatic tissue rather than prospectively validating primary-tumor scores as predictors of later metastatic response [105,114]. Primary-tumor TDS and *MAPK*-output scores should therefore remain research biomarkers until paired primary–metastatic and lesion-level prospective studies establish predictive validity.

### 8.2. Single-Cell, Spatial, and Computational Resolution of Functional Differentiation

Bulk transcriptomic measurements can obscure distinct malignant subpopulations and microenvironmental interactions within tumors. Single-cell RNA sequencing and spatial transcriptomics offer complementary methods to address this heterogeneity. Recent TC studies reveal significant diversity among malignant thyrocytes and their microenvironment, mapping cellular states during progression from differentiated to advanced disease [125,126]. These techniques could identify reversible and fixed states of lineage suppression in lesions and highlight niches that limit *SLC5A5/NIS* restoration after *MAPK* inhibition, although their predictive value for redifferentiation response needs further validation [125,126].

Artificial intelligence (AI) and machine learning may help integrate molecular, imaging, histopathologic, and dosimetric data. A recent deep-learning framework successfully classified TC differentiation states using combined molecular and metabolic features [127]. However, there is currently no validated AI model for patient selection for redifferentiation therapy, and future models require external validation to demonstrate predictive value beyond existing clinical factors.

### 8.3. Biomarker-Guided Combination and Trial Design

Combination strategies should be driven by demonstrated resistance biology rather than empiric escalation. Adaptive *HER2/HER3*-mediated *MAPK* rebound may support receptor-family cotargeting, persistent *PI3K–AKT* signaling may justify dual-pathway inhibition, and persistent chromatin repression may identify tumors in which epigenetic cotargeting warrants investigation [69,96,99]. Driver-specific strategies should also be evaluated separately because *BRAF-, RAS-, RET*-, and *NTRK*-driven tumors differ in baseline differentiation and pathway biology [16,17,128]. Crucially, future trials should include designs capable of separating the direct antitumor activity of kinase inhibition from the incremental benefit of subsequent ^131I, particularly now that randomized evidence demonstrates clinically meaningful direct *BRAF/MEK* activity in *BRAF^V600E*-positive RAIR-DTC [129].

### 8.4. Standardization of Functional Imaging and Dosimetry

Standardization of functional imaging and dosimetry is equally important. Existing redifferentiation studies differ in radionuclide imaging, criteria for defining restored avidity, administered ^131I activity, and absorbed-dose thresholds [38,105,114]. ^124I PET/CT offers quantitative lesion-level assessment where available, but a universally validated absorbed-dose threshold defining clinically meaningful redifferentiation has not been established. Recent lesion-level analyses from redifferentiation cohorts further emphasize substantial interlesional variability in delivered radiation dose and its relationship with tumor response [130]. Future multicenter trials should prospectively report lesion-level uptake, iodine residence time, predicted and post-therapeutic absorbed dose, interlesional heterogeneity, and subsequent structural response using harmonized methodology rather than relying solely on visual reacquisition of iodine avidity [114,130].

The critical endpoint for the field is therefore not restoration of iodine uptake alone, but identification of patients in whom redifferentiation produces a sufficiently durable and dosimetrically meaningful restoration of RAI sensitivity to improve clinically relevant outcomes.

## 9. Conclusions

The progression of differentiated thyroid carcinoma into a poorly differentiated, radioiodine-refractory state is fundamentally tethered to the oncogenic hijacking of the *MAPK/ERK* signaling axis. This review synthesizes current evidence showing that in the context of thyroid cancer, *ERK1/2* functions not merely as a dominant mitogenic switch but as a central epigenetic and transcriptional orchestrator of lineage suppression. The synthesis of current preclinical and clinical data highlights several core biological paradigms. First, genotype dictates phenotype; the specific biochemical mechanism of *MAPK* activation dictates the amplitude of signaling, directly influencing the degree of cellular dedifferentiation and therapeutic resistance. Second, the silencing of critical thyroid-specific functional genes (most notably *SLC5A5, TPO*, and *TSHR*) is an active, pathway-dependent process. It is enforced through the coordinated antagonism of lineage-defining transcription factors (such as *PAX8* and *NKX2-1*) and the recruitment of repressive chromatin remodeling machinery. Crucially, this comprehensive molecular understanding has catalyzed a paradigm shift in the clinical management of advanced thyroid cancer. Radioiodine refractoriness can no longer be viewed as a static, irreversible endpoint of terminal dedifferentiation. Rather, it is a dynamic and pharmacologically targetable state of epigenetic plasticity. The advent of redifferentiation therapy—utilizing *MEK* and *BRAF* inhibitors to suppress oncogenic *ERK* signaling and restore iodine avidity—provides a powerful, biologically rational theranostic platform. While challenges regarding heterogeneous responses and adaptive resistance remain, the targeted reversal of *ERK*-driven dedifferentiation represents one of the most promising precision-medicine frontiers in thyroid oncology, offering a pathway to restore the efficacy of targeted radiotherapy in patients with aggressive, advanced disease.

## Figures and Tables

**Table 1:** Prospective Clinical Studies of Pharmacologic Restoration or Enhancement of Radioiodine Avidity in Differentiated Thyroid Cancer.

Study/Trial	Clinical Setting and Intervention	Sample Size	Principal Efficacy Finding	Toxicity and Critical Interpretation
Hoftijzer et al., 2009 [119]	Metastatic DTC; sorafenib 400 mg twice daily for 26 weeks; RAI reinduction was the primary endpoint	31	No reinduction of RAI uptake at metastatic sites. Nevertheless,8 patients (25%) achieved PR and 11 (34%) SD, demonstrating antitumor activity without restoration of iodine avidity.	No unexpected toxicity signal was reported. Important negative redifferentiation study, separating tumor control from restoration of RAI competence.
Ho et al., 2013; NCT00970359 [38]	RAIR advanced thyroid cancer; selumetinib → ^124I PET dosimetry → dosimetry-selected ^131I	20 evaluable	^124I uptake increased in 12/20 (60%); 8 met the dosimetry threshold and received ^131I; 5 PR and 3 SD. All 5 *NRAS*-mutant patients qualified for ^131I.	No grade ≥ 3 toxicity was attributed to selumetinib. Landmark proof-of-principle study, but small and nonrandomized.
Rothenberg et al., 2015; NCT01534897 [120]	*BRAF^V600E* RAIR PTC; dabrafenib → diagnostic ^131I scan → ^131I therapy if new uptake	10	New RAI uptake in 6/10 (60%); all 6 received ^131I; 2 PR and 4 SD at 3 months.	One cutaneous squamous-cell carcinoma; no other significant AEs attributed to dabrafenib. Small BRAF-selected proof-of-concept study.
Dunn et al., 2019; NCT02145143 [105]	*BRAF^V600E* RAIR thyroid cancer; vemurafenib → ^124I PET lesional dosimetry → ^131I	12 enrolled; 10 evaluable	New/increased avidity in 6/10; 4/10 met the predefined dosimetry threshold and received ^131I; at 6 months,2 PR and 2 SD.	Grade 3 toxicities included rash and palmar–plantar erythrodysesthesia; no grade 4–5 vemurafenib toxicity. The study also showed tumor regression from vemurafenib alone, highlighting confounding by direct antitumor activity.
Tchekmedyian et al., 2022; NCT02456701 [121]	*BRAF^V600E* RAIR thyroid cancer; vemurafenib + anti-ErbB3 antibody CDX-3379 → ^131I	7 enrolled; 6 evaluable	Increased RAI uptake in 5/6; 4 received ^131I; at 6 months,2 PR and 2 PD among treated patients.	No grade 3–4 toxicity was attributed specifically to CDX-3379. Very small exploratory study testing prevention of adaptive *HER3*-mediated *MAPK* rebound.
ASTRA, Ho et al., 2022; NCT01843062 * [118]	Randomized phase III; selumetinib + adjuvant ^131I vs placebo + ^131I in high-risk DTC	233 randomized	18-month CR 40% vs 38%; OR 1.07; *p* = 0.8205. No improvement in the primary endpoint.	Treatment-related grade ≥ 3 AEs occurred in 16% vs 0%. Important negative trial showing that *MAPK* inhibition should not be generalized to unselected adjuvant RAI settings.
ERRITI, Weber et al., 2022; NCT04619316 [102]	*BRAF-WT*: trametinib; *BRAF^V600E*: dabrafenib + trametinib → ^131I after successful redifferentiation	20	Successful redifferentiation in 7/20 (35%): 5/14 BRAF-WT and 2/6 *BRAF*-mutant. After ^131I: 1 PR,5 SD,1 PD.	One grade 3 pyrexia and one grade 4 rash. Baseline FDG SUV_peak < 10 was associated with successful redifferentiation.
SEL-I-METRY, Wadsley et al., 2023; ISRCTN17468602 [122]	Multicenter phase II; selumetinib → ^123I SPECT/CT assessment → ^131I in patients with sufficient uptake	30 recruited; 28 treated	11 demonstrated sufficient/increased uptake, but only 9/28 (32.1%) were clinically approved for and received ^131I; 12-month PFS in the ^131I-treated cohort was 64.8%.	Worst CTCAE toxicity was grade 3 in 13/28 (46.4%) and grade 4 in 1/28 (3.6%). Lower-than-anticipated resensitization and recruitment difficulties limited the study.
MERAIODE–BRAF, Leboulleux et al., 2023; NCT03244956 [104]	*BRAF^V600E* RAIR DTC; dabrafenib + trametinib → fixed-activity ^131I	24 enrolled; 21 evaluable at 6 months	Abnormal uptake was present in 5% at baseline diagnostic scanning,65% after targeted therapy, and 95% on post-therapy scanning. At 6 months: 38% PR,52% SD,10% PD; estimated 24-month PFS 68%.	AEs occurred in 96%; 10 grade 3–4 events occurred in 7 patients. Strong prospective signal, but absence of a control arm prevents separation of targeted-therapy and ^131I effects.
MERAIODE–RAS, Leboulleux et al., 2023; NCT03244956 [103]	*RAS*-mutant metastatic RAIR DTC; trametinib followed by ^131I	11 enrolled; 10 received/evaluable for ^131I outcome	At 6 months,2/10 (20%) PR,7/10 (70%) SD, and 1/10 (10%) PD. Restoration/enhancement of iodine avidity did not translate into a high structural-response rate.	Toxicity was predominantly manageable, but treatment-related AEs occurred. Particularly important for demonstrating that increased iodine avidity and objective tumor response are not equivalent endpoints.
RESET, Dotinga et al., 2025; NCT04858867 [123]	Prospective phase II; RAIR-DTC requiring lenvatinib; ^124I PET/CT dosimetry at baseline and after 6/12 weeks of lenvatinib	9	0/9 became eligible for ^131I using the prespecified ≥ 20-Gy predicted lesion-dose criterion. Despite this, lenvatinib produced overall PR in 4/9 patients and target-lesion PR rates of 59% at week 6 and 75% at week 12.	Dose reduction in 5/9; temporary interruption in 2/9; grade 3 hypertension in 7/9 and one grade 4 dyspnea SAE. Particularly strong evidence that direct tumor response does not imply redifferentiation.

AE, adverse event; BRAF-WT, BRAF wild-type; CR, complete response; DTC, differentiated thyroid carcinoma; FDG, ^18F-fluorodeoxyglucose; HR, hazard ratio; MEK, mitogen-activated protein kinase kinase; PD, progressive disease; PET, positron emission tomography; PFS, progression-free survival; PR, partial response; PTC, papillary thyroid carcinoma; RAI, radioactive iodine; RAIR, radioiodine-refractory; RECIST, Response Evaluation Criteria in Solid Tumors; SD, stable disease; SPECT/CT, single-photon emission computed tomography/computed tomography; SUV_peak, peak standardized uptake value; TKI, tyrosine kinase inhibitor. * ASTRA was conducted in patients with high-risk DTC receiving adjuvant RAI rather than established RAIR disease and is included because it provides randomized evidence regarding pharmacologic enhancement of RAI efficacy.

## Data Availability

No new data were created or analyzed in this study. Data sharing does not apply to this article.

## Abbreviations

AE	adverse event
AI	artificial intelligence
AKT	protein kinase B
ATA	American Thyroid Association
ATC	anaplastic thyroid carcinoma
BRAF	B-Raf proto-oncogene, serine/threonine kinase
BRAF-WT	BRAF wild-type
cAMP	cyclic adenosine monophosphate
ceRNA	competing endogenous RNA
circRNA	circular RNA
CR	complete response
CT	computed tomography
CTCAE	Common Terminology Criteria for Adverse Events
DDR1	discoidin domain receptor tyrosine kinase 1
DIO1/2	iodothyronine deiodinase 1/2
DNMT1	DNA methyltransferase 1
DTC	differentiated thyroid carcinoma
DUSP5	dual-specificity phosphatase 5
DUOX1/2	dual oxidase 1/2
EGF	epidermal growth factor
EGFR	epidermal growth factor receptor
ELK1	ETS transcription factor ELK1
EMT	epithelial–mesenchymal transition
ERK	extracellular signal-regulated kinase
EZH2	enhancer of zeste homolog 2
FDG	^18F-fluorodeoxyglucose
FOS	Fos proto-oncogene/AP-1 transcription factor subunit
FOXE1	forkhead box E1
FOXM1	forkhead box M1
FTC	follicular thyroid carcinoma
GABP	GA-binding protein
HDAC	histone deacetylase
HER2/3	human epidermal growth factor receptor 2/3
HHEX	hematopoietically expressed homeobox
HR	hazard ratio
IGF-I	insulin-like growth factor I
IHC	immunohistochemistry
ITOG	International Thyroid Oncology Group
JAK–STAT	Janus kinase–signal transducer and activator of transcription
lncRNA	long non-coding RNA
MAPK	mitogen-activated protein kinase
MEK	mitogen-activated protein kinase kinase
METTL3	methyltransferase-like 3
miRNA	microRNA
mRNA	messenger RNA
mTOR	mechanistic target of rapamycin
m6A	N6-methyladenosine
ncRNA	non-coding RNA
NIS	sodium/iodide symporter
NKX2-1	NK2 homeobox 1
NTRK	neurotrophic receptor tyrosine kinase
OR	odds ratio
PAX8	paired box 8
PD	progressive disease
PDTC	poorly differentiated thyroid carcinoma
PET	positron emission tomography
PFS	progression-free survival
PI3K	phosphoinositide 3-kinase
PR	partial response
PRC2	polycomb repressive complex 2
PTC	papillary thyroid carcinoma
RAI	radioactive iodine
RAIR	radioiodine-refractory
RAIR-DTC	radioiodine-refractory differentiated thyroid carcinoma
RARβ2	retinoic acid receptor beta 2
RAS	RAS family of small GTPases
RET	RET proto-oncogene
RTK	receptor tyrosine kinase
SAE	serious adverse event
SAHA	suberoylanilide hydroxamic acid
SD	stable disease
SHC	Src homology 2 domain-containing adaptor protein
SLC5A5	solute carrier family 5 member 5
SLC26A4	solute carrier family 26 member 4
SMAD3	SMAD family member 3
SPECT/CT	single-photon emission computed tomography/computed tomography
SWI/SNF	switch/sucrose non-fermentable
TC	thyroid cancer
TCGA	The Cancer Genome Atlas
TDS	thyroid differentiation score
TERT	telomerase reverse transcriptase
Tg	thyroglobulin
TGF-β	transforming growth factor beta
TKI	tyrosine kinase inhibitor
TPO	thyroid peroxidase
TP53	tumor protein p53
TSH	thyroid-stimulating hormone
TSHR	thyroid-stimulating hormone receptor
TTF-1	thyroid transcription factor 1 (NKX2-1)
TTF-2	thyroid transcription factor 2 (FOXE1)
TWIST1	twist family basic helix–loop–helix transcription factor 1
USP15	ubiquitin-specific peptidase 15
